# Evaluation of the Relationship between Early Troponin Clearance and Short-Term Mortality in Patients with Chronic Renal Failure

**DOI:** 10.1155/2020/6328037

**Published:** 2020-01-31

**Authors:** Ahmet Ozbek, Abdullah Algın, Gokhan Tas, Mehmet Ozgur Erdogan

**Affiliations:** ^1^Department of Emergency Medicine, University of Health Sciences Sisli Etfal Training and Research Hospital, Istanbul, Turkey; ^2^Department of Emergency Medicine, University of Health Sciences Umraniye Training and Research Hospital, Istanbul, Turkey; ^3^Department of Emergency Medicine, Sakarya University Training and Research Hospital, Adapazarı, Sakarya, Turkey; ^4^Department of Emergency Medicine, Bahcesehir University, Istanbul, Turkey

## Abstract

**Objective:**

In patients with CKD, cTn concentrations may be elevated in the absence of AMI, which is a predicted finding caused by chronic structural heart disease rather than acute injury. The increase in troponin level observed in noncardiac conditions provides conflicting results when predicting mortality. Low lactate clearance was associated with increased mortality. Lactate clearance is calculated as follows: (early lactate − late lactate/early lactate) *∗* 100. We aimed to investigate whether troponin clearance calculated according to this formula had an effect on short-term mortality.

**Methods:**

The study included 300 patients with chronic renal failure who had a sepsis-related organ failure assessment (SOFA) score ≥3. By taking the baseline troponin at the time of emergency presentation as reference and comparing them with the fourth-hour troponin values, troponin clearance was investigated in the evaluation of mortality among hospitalized patients with CKD within the first month after discharge. The data obtained were analyzed using the SPSS data analysis software version 20.0. Student's *t*-test was used for the parametric data, and the Chi-squared test for the nonparametric data.

**Results:**

Of the 300 patients evaluated, 189 patients survived (mean age 66.20 ± 14.597 years), and 111 died (mean age 74.81 ± 12.916 years). Troponin clearance was detected in 40 of the 111 patients in the mortality group and 119 of the 189 patients in the survival group. Troponin clearance was significantly more frequent in surviving patients (*P*=0.0000083).

**Conclusion:**

Troponin clearance can be considered as a valuable leading indicator of survival, but higher levels of troponin clearance did not lead to higher survival rates.

## 1. Introduction

Cardiac troponin I (cTnI) and cardiac troponin T (cTnT), which are sensitive biological markers of myocardial injury, are used to diagnose acute myocardial infarction (AMI) [1–4]. Although cardiac troponin (cTn) testing is quite specific in detecting myocardial damage, multiple diseases, including chronic kidney disease (CKD), may lead to injury that increases cTn [1–8]. In patients with CKD, cTn concentrations may be elevated in the absence of AMI [8], which is a predicted finding caused by chronic structural heart disease rather than acute injury [5, 6].

Increased cTn has been observed in the absence of acute coronary syndrome (ACS) in both cardiac and noncardiac patients. There are additional questions concerning the causes of elevated cTn and the reasons for the observed differences between the increases in cTnT and cTnI. To answer these questions, it may be helpful to examine the synthesis, susceptibility, destruction, and/or clearance process of cTn [9].

The association between elevated cTnT and low glomerular filtration rate (eGFR) is stronger compared to that of cTnI to eGFR [10, 11], suggesting that cTnT clearance is renal, while cTnI clearance is nonrenal [12, 13]; however, there is not sufficient evidence to support this idea. Only one study reported cTnT in urine in patients with AMI; however, no cTnI was detected. In that study, it was shown that greater molecular weight (kDa) was associated with cTn accumulation in the presence of impaired renal function [14]. Contradicting this theory are the results of a recent study, which indicate a marked increase in cardiac biomarkers in cases presenting with low eGFR and low molecular weight (<25 kDa). These contradictory results suggest that there are still unanswered questions concerning the clearance of cTn.

Some emergency departments and intensive care units use serum lactate concentration as a qualitative indicator of resuscitation in patients with sepsis. Bolvardi et al. revealed that low lactate clearance was associated with increased mortality; however, direct lactate elevation yielded contradictory results as a predictor of mortality [15]. Similarly, the increase in troponin observed in noncardiac conditions provides conflicting results in predicting mortality. Lactate clearance is calculated as follows: (early lactate − late lactate/early lactate) *∗* 100 [16]. In the current study, we aimed to investigate whether troponin clearance calculated according to this formula had an effect on short-term mortality.

## 2. Materials and Methods

Patients who presented to the Emergency Department of Haydarpasa Numune Training and Research Hospital with CKD between January 2013 and December 2015 and scored ≥3 in the sequential (sepsis-related) organ failure assessment (SOFA) were retrospectively analyzed using the hospital data screening system. Of the 6,000 patients with CKD, a total of 300 (167 males and 133 females) were included in the study.

By taking the baseline troponin at the time of emergency presentation as reference and comparing them with fourth-hour troponin values, troponin clearance was investigated in the evaluation of mortality among hospitalized patients with CKD within the first month after discharge. Serum cTnT levels were measured using the dimension analyzer (Dade Behring Diagnostic, Amersfoort, Netherlands) through application of the one-step sandwich principle immunoassay.

The patients were analyzed in two ways; firstly, they were analyzed in terms of presence or absence of measurable troponin clearance. Patients with a baseline level of troponin higher than their subsequently measured 4^th^ hour troponin were deemed measurable. Conversely, patients with a 4^th^ hour troponin level higher than baseline levels were deemed to have no measurable troponin clearance. In patients with measurable troponin clearance, the mean value of their respective groups' clearances was analyzed as well. The parameters analyzed from the records were age, gender, blood urea nitrogen (BUN), creatinine, baseline and fourth-hour troponin values, and mortality within one month of discharge from hospital.

Patients were divided into two groups based on their mortality or survival. Patients who died within a month of admission were assigned to the mortality group, while surviving patients were assigned to the survivor group. The inclusion criteria were as follows: presence of stages 4 and 5 CKD, an increase in troponin due to non-ACS causes, being above 18 years of age, a SOFA score of ≥3, and availability of data regarding first-month mortality. The patients with missing files, unknown troponin levels or ACS, those who died in the emergency department before examination, those with additional diseases that might cause elevated troponin, and those who were referred to the hospital from another health center were excluded.

### 2.1. Statistical Analysis

The data obtained were analyzed using the SPSS data analysis software version 20.0. Student's *t*-test was used for the parametric data and the Chi-squared test for the nonparametric data. *P* < 0.05 was considered statistically significant.

## 3. Results

Of the 300 patients evaluated, 189 patients survived (mean age: 66.20 ± 14.597 years), and 111 died (mean age: 74.81 ± 12.916 years). There was a statistically significant difference between the two groups in terms of mean age (*P* < 0.05). Of the survivors, 84 were female, and 105 were male; while there were 50 female patients and 61 male patients in the mortality group. There was no statistically significant relationship between gender and mortality (*P*=0.507).

A statistically significant difference was observed between the mortality and survivor groups in terms of the relationship between age and mortality, but no statistically significant difference was found in relation to renal function or baseline and fourth-hour mean troponin values between groups (Table 1).

Measurable troponin clearance was detected in 40 of the 111 patients in the mortality group and 119 of the 189 patients in the survival group. Troponin clearance was significantly more frequent in surviving patients (*P*=0.0000083) (Table 2).

Creatinine clearance was calculated as 19.73 ± 9.47 in patients without troponin clearance and 21.44 ± 12.2 in those with troponin clearance, indicating no statistically significant correlation (*P*=0.174) (95% confidence interval, CI = −4.18–0.75). Of the 159 patients with troponin clearance, 119 survivors had a mean troponin clearance of 31.32 ± 42.90, while the 40 who died had mean troponin clearance of 24.38 ± 21.11. There was no significant difference between the mortality and survival groups in terms of the mean troponin clearance values (*P*=0.156) (Table 3), i.e., a higher mean value of troponin clearance was not associated with a higher rate of survival.

## 4. Discussion

A large number of studies have shown that in patients with end-stage renal disease (ESRD), both cTnI and cTnT are strong prognostic indicators for death of all causes and although cTnI increases less than cTnT, it is associated with a higher risk of mortality [17–19].

In a study by Alcalai et al., the cTnT levels were measured in all patients who presented to the hospital for various reasons within a 10-month period. Of the 635 cases with a cTnT value of >0.1 *μ*g/l, 53% were diagnosed with ACS and 41% of these cases were associated with nonthrombotic causes (nonischemic cardiac events, such as myocarditis and arrhythmia in 5%, sepsis in 8%, surgery in 5%, renal failure in 2%, and cardiopulmonary resuscitation in 2%). For the remaining 6% of patients, the reason for the elevated cTnT could not be determined. The results of that study show that although a significant proportion of the patients admitted to hospital had high cTn levels, this was not due to coronary artery disease [20].

Since coronary ischemia is frequently present in patients with renal failure, it is important to ascertain whether the cTn elevation is caused by ischemia or chronic progression of impaired renal function. Although there is no specific method to appropriately make this distinction, increased cTn levels in follow-up are mostly interpreted in favor of acute ischemia [21]. In patients included in the study of the Global Use of Strategies to Open Occluded Coronary Arteries IV, it was reported that elevated cTn was an important predictor of short-term prognosis regardless of creatinine clearance [22].

Increased cTn, in the absence of ACS, is observed in patients with both cardiac and noncardiac conditions. This raises additional questions concerning the causes of elevated cTn and the observed differences between the increases in cTnT and cTnI. To answer these questions, it may be helpful to examine the synthesis, susceptibility, destruction, and/or clearance process of cTn [9]. To the best of our knowledge, there are no publications focusing on the clinical value of the follow-up troponin course in patients with a noncardiac troponin increase. The value of troponin clearance in surveying has not been investigated.

Life-threatening complications of renal failure, such as pulmonary edema associated with fluid overload, hyperkalemia, other electrolyte and acid-base disorders, increased acute coronary frequency, dyslipidemias, and chronic volume overload, also increase the cardiovascular burden, leading to an imbalance in myocardial oxygen consumption, coronary vasoconstriction, and myocardial injury [23]. Continuation of this condition will result in a significant increase in troponin values and nonclearance of troponin. Effective treatment and successful management of the disease can reduce hypoxia-related myocardial perfusion disorder and reduce troponin values during follow-up. This was found to be significantly associated with survival in our study.

It is important to acknowledge the potential limitations of the current study. As a single-center retrospective study, the effects of unrecorded factors that may have led to confusion cannot be denied. Furthermore, the exact etiology of CKD may not have been documented by the treating clinical team in all cases.

In conclusion, in this study, measurable troponin clearance was shown to be associated with increased probability of survival in critical cases, but a higher mean value of troponin clearance did not lead to a higher survival rate. According to the overall results, presence of measurable troponin clearance may be valuable as a leading indicator of survival; however, for troponin clearance to be used as an independent predictor of survival, our results should also be confirmed in patient groups with conditions other than CKD. For this purpose, multicentered studies involving larger case series are needed.

## Figures and Tables

**Table 1:** Age, renal function, and initial and fourth-hour troponin values in mortality and survivor groups.

	Mortal:1 Survivor:0	*N*	Mean	Std. deviation	*P*
Age (years)	0	189	66.20	14.597	*P* < 0.05
	1	111	74.81	12.916	

Creatinine (mg/dL)	0	189	4.2689	2.66015	*P* : 0.842
	1	111	4.2107	2.28692	

BUN (mg/dL)	0	189	65.38	28.427	*P* : 0.326
	1	111	68.80	29.471	

Initial troponin (ng/mL)	0	189	0.22262	0.216311	*P* : 0.891
	1	111	0.22617	0.217616	

4^th^ hour troponin (ng/mL)	0	189	0.20530	0.200472	*P* : 0.031
	1	111	0.25965	0.213819	

**Table 2:** Evaluation of the relationship between survival and measurable troponin clearance.

	Clearance		
	No troponin clearance	Troponin clearance	Total
Survivor	70	119	189
Exitus	71	40	111
Total	141	159	300

**Table 3:** Distribution of patients with troponin clearance.

	Survivor:0 Exitus:1	*N*	Mean	Std. deviation	95% CI	
					Lower	Upper
Troponin clearance	0	119	31.32	42.90	−7.014	20.89
	1	40	24.38	21.11	−3.262	17.14

## Data Availability

SPSS/Excel was used for data storage. Data are available from Mehmet Özgür Erdoğan, Department of Emergency Medicine, Bahcesehir University, Istanbul, Turkey, for researchers who meet the criteria for access to confidential data (ozgurtheerdogan@mynet.com).
